# Palatal Soft Tissue Thickness on Maxillary Posterior Teeth and Its Relation to Palatal Vault Angle Measured by Cone-Beam Computed Tomography

**DOI:** 10.1155/2020/8844236

**Published:** 2020-09-09

**Authors:** Doosadee Hormdee, Thanwarat Yamsuk, Pipop Sutthiprapaporn

**Affiliations:** ^1^Division of Periodontology, Department of Oral Medical Science, Faculty of Dentistry and Research Group of Chronic Inflammatory Oral Diseases and Systemic Diseases Associated with Oral Health, Khonkaen University, Khon Kaen 40002, Thailand; ^2^Department of Periodontology, Faculty of Dentistry, Khonkaen University, Khon Kaen 40002, Thailand; ^3^Division of Orthodontic, Department of Preventive Dentistry, Faculty of Dentistry, Khonkaen University, Khon Kaen 40002, Thailand

## Abstract

**Objective:**

Analyzing palatal soft tissue thickness in cone-beam computed tomography (CBCT) images and evaluating the relationship between tissue thickness and palatal vault angulation.

**Methods:**

Out of 1,737 CBCT images, fifty-six images met the inclusion criteria and were included in this cross-sectional study. The palatal vault angle on the maxillary first molar was measured and divided the images into 3 groups. The soft tissue thickness between the maxillary first premolar and second molar was measured at a distance of 3, 6, 7, 8, and 9 mm from the cementoenamel junction. All the image measurements were performed using CBCT-viewer software.

**Result:**

In this study, 56 CBCT images with full permanent maxillary posterior teeth and absence of light scattering were found. The mean age of the patients was 31.59 ± 13.92 years. The moderate and deep palatal vault angle patterns had the greatest and least prevalence, respectively. The average thickness on shallow, moderate, and deep palatal vault groups was 4.02 ± 0.58, 3.75 ± 0.73, and 3.43 ± 0.38 mm, respectively. Furthermore, the mean palatal mucosal thickness was statistically different between the deep and shallow palatal vault angle groups (*p* < 0.05, power of test 0.8). Based on the Pearson correlation coefficient, there was a negative correlation between the palatal mucosal thickness and palatal vault angle (*p* < 0.05, power of test 0.85).

**Conclusion:**

A negative correlation between the palatal mucosal thickness and palatal vault angle was observed. Furthermore, this study suggested that the shape of the palatal vault can be one of the supporting data for evaluating the graft dimensions.

## 1. Introduction

Autologous connective tissue is an essential therapeutic tool in mucogingival periodontal surgery from a functional and esthetical perspective [1–4]. Harvesting of palatal donor tissues from the region between canine and second molar has now become a standard procedure [5, 6]. In most mucogingival periodontal surgeries, the quantity of palatal masticatory mucosa can dictate the treatment plan and affect the surgical outcome [1–3].

Along with variations in vital structures and palatal vault anatomy, the maximum dimensions of soft tissues that can be harvested, in terms of height and length, have been previously reported [5, 7, 8] Similarly, several methods have been described to measure the thickness of the palatal gingival tissues. Transgingival probing is a commonly performed direct method but is invasive in nature and causes considerable patient discomfort, requiring local anesthesia [9]. Ultrasound, one of the indirect measurements, is an effective method to measure gingival thickness, but it lacks reproducibility and has difficulty in obtaining a panoramic view of the periodontal structures [2]. Cone-beam computed tomography (CBCT) has been increasingly used in the maxillofacial region [10–14]. However, in previous investigations utilizing CBCT, it was difficult to establish the relationship between the form of palatal vault and the thickness of the harvesting graft. Additionally, the soft tissue thickness encompassing the entire width of the donor graft has not been well documented in the literature. The aim of this study was to evaluate the palatal mucosa thickness and its relationship with the palate vault angle using CBCT.

## 2. Material and Methods

### 2.1. Sample Selection

The review of patients' CBCT images was approved by the University Ethical Board (protocol number HE592036). Patients who had lost posterior teeth or had a pathology in the palatal region during the time of the study were excluded. Out of 1,737 CBCT images taken from March 2012 until December 2016, fifty-six images with full permanent maxillary posterior teeth and absence of scatter artifacts were included in the study.

### 2.2. Measurement of Palatal Soft Tissue Thickness

**Figure 1:**
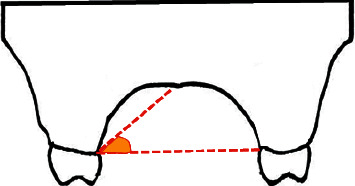
Palatal vault angle on the maxillary first molar was measured using the junction angle between the horizontal plane at the cementoenamel junction (CEJ) and an imaginary line from the midpalatal suture to the CEJ.

**Figure 2:**
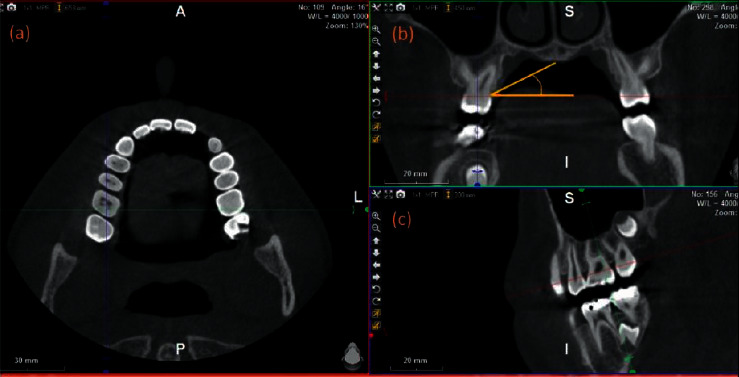
(a) A specific section on the midpalatal root of the maxillary first molar in the horizontal plane. (b) Palatal vault angle in the frontal plane. (c) A midsection of the palatal root in the sagittal plane.

**Figure 3:**
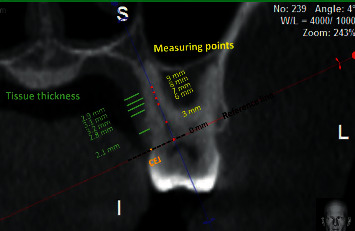
A specific section on the midpalatal root of a maxillary first molar in the frontal plane has been set. On each tooth, the palatal soft tissues at a distance of 3, 6, 7, 8, and 9 mm from CEJ were marked for measurement.

Cone-beam computed tomography was performed using the WhiteFox cone-beam 3D system (WhiteFox, WhiteFox Imaging, Italy). The technical parameters for image acquisition were 105 kV, 9 mA, field of view in 150 mm × 130 mm (full arch), and voxel size of 0.3 mm^3^. During the CBCT imaging, patients were stood so that the Frankfort plane was parallel to the floor with sagittal plane perpendicular. Measurements of the CBCT images were performed digitally using the WhiteFox imaging software version 3.0. All constructions and measurements were executed on a Samsung computer with a graphic card (NVDIA GeForce GT330M Series) and 14.1-inch Generic PnP Monitor with a resolution of 1,366 × 768 pixels and the zoom level of 150%. A specific section on the midpalatal root of the maxillary first molar was created by rotating images in 3D direction. Then, the palatal vault angle on the maxillary first molar was measured in coronal images using the junction angle between the horizontal plane at the cementoenamel junction (CEJ) and a line drawn from midpalatal suture (Figure 1). According to the palatal vault angle on specific sections of the maxillary first molar, the images were divided into 3 groups: shallow group (Group S) in which the angle was <30 degrees, moderate group (Group M) with the angle between 30 and 40 degrees, and deep group (Group D) in which the angle was >40 degrees (Figure 2). Firstly, the soft tissue thickness between the maxillary first premolar and second molar was measured. Secondly, a cross-sectional section passing through the center of the palatal root canal of the first molar was used as the reference for performing the image. The reference line passing through the CEJ on the palatal side of the first molar is perpendicular to the line passing through the palatal root canal. The reference dots marked at the palatal root were 3, 6, 7, 8, and 9 mm from the reference line. Then, the palatal soft tissue thickness was measured in each tooth parallel to the reference line using measurement tools in the software (Figure 3).

### 2.3. Statistical Analysis

Data were analyzed using IBM SPSS software version 19.0 (SPSS, Chicago, IL, USA). Student's *t*-test was used to evaluate the normality of data distribution between the two groups, whereas the Mann–Whitney *U* test was used when the investigated data were not normally distributed. The Kruskal–Wallis test with post hoc corrections was used for comparison among the groups. Pearson's correlation coefficient was employed to evaluate correlations between palatal soft tissue thickness and palatal vault angle. *p* < 0.05 was considered as statistically significant and provided power of test ≥0.8.

## 3. Result

### 3.1. Demographic Characteristics of the Study Population

The demographic characteristics of the three investigated groups are as shown in Table 1. A total of 56 CBCT images that met the inclusion criteria were obtained from 1737 consecutive patients from University Hospital, and included 30 women and 26 men, with a mean age of 31.59 years. There were no statistically significant differences in gender and age between the groups.

### 3.2. Thickness of Palatal Soft Tissue

The mean soft tissue thickness of the donor area for all patients was 3.71 ± 0.65 mm, ranging from 2.14 to 5.27 mm. The mean palatal soft tissue thickness of each posterior tooth is described as range and mean ± SD in Table 2, whereas palatal soft tissue thickness at 5 different points is shown in Table 3. The average soft tissue thickness from shallow, moderate, and deep palatal vault angles was 4.02, 3.75, and 3.43 mm, respectively. No statistically significant differences were found between the groups, but thicker palatal soft tissue was observed in the lower palatal vault angle for all the teeth that were measured. Furthermore, it was also revealed that the further the distance from CEJ, the thicker the palatal soft tissue, as shown in Table 3.

### 3.3. Correlations between Palatal Vault Angle and Thickness of Palatal Soft Tissue

**Figure 4:**
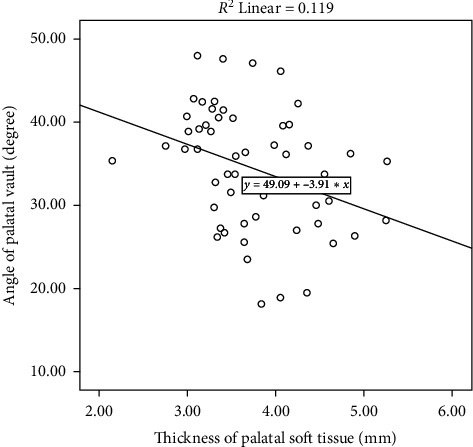
Scatter plot showing the correlation between the palatal vault angle and thickness of palatal soft tissue (correlation coefficient = −0.345, *p* value = 0.010).

The correlations between the angle of palatal vault and thickness of palatal soft tissue are as presented in Figure 4. Pearson correlation analysis, determined from the entire subjects, showed significantly negative correlations between the vault angle and soft tissue thickness (correlation coefficient = −0.345, *p*=0.010).

## 4. Discussion

The palatal masticatory mucosa used as a connective tissue donor site in plastic periodontal surgery is reported to have high success rates [1, 15, 16]. Previous assessment of graft dimensions at the palatal donor site reported the maximum harvestable height and width of the soft tissue in relation to the location of the greater palatine artery [6, 10, 17]. For functional and esthetic concerns, a minimum dimension of 5 mm is required to cover shallow recessions [3]. Studies on the dimension of palatal graft have revealed that 5 mm wide connective tissue can be harvested from the premolar area in all cases [10, 17]. On the other hand, Reiser et al. proposed to subdivide the palatal vault into three groups—high, average, and shallow palatal vaults. Correlations between the distance of CEJ to the greater palatine artery and the type of palatal vault have also been reported. Precautionary measures should be taken when dealing with shallow palatal vault in order to prevent damage to the greater palatine artery [6]. Kim et al. showed that palatal soft tissue thickness increased gradually from the CEJ toward the apical region and surgical placement of miniscrew for orthodontic anchorage requires consideration of the placement site and angle based on anatomical characteristics [18]. However, this article emphasized the interdental area measurements, which is different from our study. The results of this study were in concordance with a study by Yilmaz et al., which reported that the thickest palatal masticatory mucosa was from the second premolar and the thinnest was from the first molar [11]. This difference may be explained by the anatomical variations of the palatal root of the first molar, which can act as an obstacle in graft harvesting. According to the negative correlation between the thickness of palatal masticatory mucosa and the degree of palatal vault observed in this study, it can be suggested that patients with steep angle have thinner harvestable palatal tissue.

## 5. Conclusion

The harvesting of palatal graft may be limited by anatomical landmarks and tissue thickness. This study suggested that the shape of the palatal vault can be one of the supporting data for evaluating the graft dimension. The operating surgeon needs to exercise caution when dealing with patients with shallow palatal vault as the donor graft can be deficient in the length, whereas, in patients with steep palatal vault, the harvested tissues may be undesirably thick.

## Figures and Tables

**Table 1:** Demographic data of the study groups.

	Total (*n* = 56)	Group S (*n* = 17)	Group M (*n* = 26)	Group D (*n* = 13)	*p* value
Gender					
Male	26 (46.40 %)	9	11	6	0.801
Female	30 (53.60%)	8	15	7	
Age					
Mean ± SD	31.59 ± 13.92	37.12 ± 15.52	31.04 ± 12.92	25.46 ± 11.59	0.070
Range	14–59	14–59	14–56	15–48	

**Table 2:** Palatal tissue thickness data of all posterior teeth from 3 different palatal vault angle types.

	Total	Group S	Group M	Group D	*p* value
Maxillary first premolar					
Mean ±SD	3.90 ± 0.73	4.13 ± 0.82	3.92 ± 0.76	3.57 ± 0.36	0.113
Range	2.38–6.47	3.14–6.47	2.38–5.51	3.11–4.39	

Maxillary second premolar					
Mean ± SD	4.38 ± 0.83	4.80 ± 0.85	4.29 ± 0.87	4.00 ± 0.40	0.021^*∗*^
Range	2.04–6.51	3.16–6.51	2.04–6.17	3.43–4.84	

Maxillary first molar					
Mean ± SD	2.78 ± 0.68	2.97 ± 0.62	2.77 ± 0.71	2.55 ± 0.65	0.248
Range	1.39–4.31	1.81–3.89	1.49–4.31	1.39–3.59	

Maxillary second premolar					
Mean ± SD	3.86 ± 1.46	4.19 ± 1.70	3.76 ± 1.50	3.61 ± 0.99	0.514
Range	1.61–8.02	1.99–7.99	1.91–8.02	2.40–6.16	

Maxillary posterior teeth					
Mean ± SD	3.71 ± 0.65	4.02 ± 0.58	3.75 ± 0.73	3.43 ± 0.38	0.036^*∗*^
Range	2.14–5.27	3.31–5.24	2.14–5.22	2.99–4.25	

^*∗*^ *p* value<0.05.

**Table 3:** Mean and standard deviation of palatal soft tissues measured at 5 different distances from CEJ.

	Group	Measuring point				
		3 mm	6 mm	7 mm	8 mm	9 mm
First premolar	S	3.19 ± 0.66	3.99 ± 0.79	4.22 ± 0.91	4.61 ± 1.08	4.62 ± 1.23
	M	3.09 ± 0.95	3.89 ± 0.70	4.03 ± 0.82	4.17 ± 0.82	4.41 ± 0.90
	D	2.71 ± 0.50	3.47 ± 0.36	3.61 ± 0.35	3.92 ± 0.56	4.13 ± 0.66

Second premolar	S	3.20 ± 0.65	4.48 ± 0.69	5.43 ± 2.26	5.18 ± 1.00	5.70 ± 1.65
	M	3.00 ± 0.78	4.19 ± 1.23	4.63 ± 0.96	4.74 ± 0.90	4.89 ± 0.97
	D	2.85 ± 0.48	3.83 ± 0.59	4.17 ± 0.58	4.48 ± 0.57	4.67 ± 0.56

First molar	S	2.21 ± 0.61	2.69 ± 0.48	3.03 ± 0.72	3.67 ± 1.00	3.67 ± 1.01
	M	2.20 ± 0.76	2.52 ± 0.85	2.79 ± 0.77	3.34 ± 0.98	3.34 ± 0.98
	D	2.33 ± 0.62	2.28 ± 0.70	2.41 ± 0.82	3.11 ± 0.72	3.12 ± 0.72

Second molar	S	2.45 ± 0.90	3.56 ± 1.71	4.18 ± 1.96	5.05 ± 1.89	5.68 ± 2.58
	M	2.66 ± 1.19	3.11 ± 1.57	3.64 ± 1.77	4.31 ± 1.94	5.07 ± 2.15
	D	2.78 ± 1.07	3.06 ± 1.04	3.34 ± 1.36	4.02 ± 1.52	4.85 ± 1.95

## Data Availability

The CBCT data used to support the findings of this study are restricted by the Khon Kaen University Hospital in order to protect patient privacy. Data are available for researchers who meet the criteria for access to confidential data.

## References

[1] Langer B., Langer L. (1985). Subepithelial connective tissue graft technique for root coverage. *Journal of Periodontology*.

[2] Eger T., Müller H.-P., Heinecke A. (1996). Ultrasonic determination of gingival thickness. Subject variation and influence of tooth type and clinical features. *Journal of Clinical Periodontology*.

[3] Monnet-Corti V., Santini A., Glise J.-M. (2006). Connective tissue graft for gingival recession treatment: assessment of the maximum graft dimensions at the palatal vault as a donor site. *Journal of Periodontology*.

[4] Langer B., Calagna L. (1980). The subepithelial connective tissue graft. *The Journal of Prosthetic Dentistry*.

[5] Anuradha B., Gopinadh A., Shankar B. S., John B., Prasad K. R. V., Devi K. N. N. (2013). Assessment of palatal masticatory mucosa: a cross-sectional study. *The Journal of Contemporary Dental Practice*.

[6] Reiser G. M., Bruno J. F., Mahan P. E., Larkin L. H. (1996). The subepithelial connective tissue graft palatal donor site: anatomic considerations for surgeons. *The International Journal of Periodontics & Restorative Dentistry*.

[7] Chaturvedi S., Khaled Addas M., Al Humaidi A. S. A., Al Qahtani A. M., Al Qahtani M. D. (2017). A novel approach to determine the prevalence of type of soft palate using digital intraoral impression. *International Journal of Dentistry*.

[8] Mustafa A. G., Tashtoush A. A., Alshboul O. A., Allouh M. Z., Altarifi A. A. (2019). Morphometric study of the hard palate and its relevance to dental and forensic sciences. *International Journal of Dentistry*.

[9] Wara-aswapati N., Pitiphat W., Chandrapho N., Rattanayatikul C., Karimbux N. (2001). Thickness of palatal masticatory mucosa associated with age. *Journal of Periodontology*.

[10] Song J.-E., Um Y.-J., Kim C.-S. (2008). Thickness of posterior palatal masticatory mucosa: the use of computerized tomography. *Journal of Periodontology*.

[11] Yilmaz H. G., Boke F., Ayali A. (2015). Cone-beam computed tomography evaluation of the soft tissue thickness and greater palatine foramen location in the palate. *Journal of Clinical Periodontology*.

[12] Januário A. L., Barriviera M., Duarte W. R. (2008). Soft tissue cone-beam computed tomography: a novel method for the measurement of gingival tissue and the dimensions of the dentogingival unit. *Journal of Esthetic and Restorative Dentistry*.

[13] Ueno D., Sekiguchi R., Morita M. (2014). Palatal mucosal measurements in a Japanese population using cone-beam computed tomography. *Journal of Esthetic and Restorative Dentistry*.

[14] Egbert N., Cagna D. R., Ahuja S., Wicks R. A. (2015). Accuracy and reliability of stitched cone-beam computed tomography images. *Imaging Science in Dentistry*.

[15] Zuhr O., Bäumer D., Hürzeler M. (2014). The addition of soft tissue replacement grafts in plastic periodontal and implant surgery: critical elements in design and execution. *Journal of Clinical Periodontology*.

[16] Prato G. P., Clauser C., Cortellini P. (1995). Periodontal plastic and mucogingival surgery. *Periodontology 2000*.

[17] Klosek S. K., Rungruang T. (2009). Anatomical study of the greater palatine artery and related structures of the palatal vault: considerations for palate as the subepithelial connective tissue graft donor site. *Surgical and Radiologic Anatomy*.

[18] Kim H.-J., Yun H.-S., Park H.-D., Kim D.-H., Park Y.-C. (2006). Soft-tissue and cortical-bone thickness at orthodontic implant sites. *American Journal of Orthodontics and Dentofacial Orthopedics*.

